# Homer1a Attenuates Endoplasmic Reticulum Stress-Induced Mitochondrial Stress After Ischemic Reperfusion Injury by Inhibiting the PERK Pathway

**DOI:** 10.3389/fncel.2019.00101

**Published:** 2019-03-15

**Authors:** Jialiang Wei, Xiuquan Wu, Peng Luo, Kangyi Yue, Yang Yu, Jingnan Pu, Lei Zhang, Shuhui Dai, Donghui Han, Zhou Fei

**Affiliations:** ^1^Department of Neurosurgery, Xijing Hospital, Fourth Military Medical University, Xi’an, China; ^2^Department of Health Services, Fourth Military Medical University, Xi’an, China; ^3^Department of Urology, Xijing Hospital, Fourth Military Medical University, Xi’an, China

**Keywords:** ischemic stroke, homer1a, mitochondrial dysfunction, endoplasmic reticulum stress, PERK kinase

## Abstract

Homer1a is the short form of a scaffold protein that plays a protective role in many forms of stress. However, the role of Homer1a in cerebral ischemia/reperfusion (I/R) injury and its potential mechanism is still unknown. In this study, we found that Homer1a was upregulated by oxygen and glucose deprivation (OGD) and that overexpression of Homer1a alleviated OGD-induced lactate dehydrogenase (LDH) release and cell death in cultured cortical neurons. After OGD treatment, the overexpression of Homer1a preserved mitochondrial function, as evidenced by less cytochrome c release, less reactive oxygen species (ROS) production, less ATP and mitochondrial membrane potential (MMP) loss, less caspase-9 activation, and inhibition of endoplasmic reticulum (ER) stress confirmed by the decreased expression of phosphate-PKR-like ER Kinase (p-PERK)/PERK and phosphate- inositol-requiring enzyme 1 (p-IRE1)/IRE1 and immunofluorescence (IF) staining. In addition, mitochondrial protection of Homer1a was blocked by the ER stress activator Tunicamycin (TM) with a re-escalated ROS level, increasing ATP and MMP loss. Furthermore, Homer1a overexpression-induced mitochondrial stress attenuation was significantly reversed by activating the PERK pathway with TM and p-IRE1 inhibitor 3,5-dibromosalicylaldehyde (DBSA), as evidenced by increased cytochrome c release, increased ATP loss and a higher ROS level. However, activating the IRE1 pathway with TM and p-PERK inhibitor GSK2656157 showed little change in cytochrome c release and exhibited a moderate upgrade of ATP loss and ROS production in neurons. In summary, these findings demonstrated that Homer1a protects against OGD-induced injury by preserving mitochondrial function through inhibiting the PERK pathway. Our finding may reveal a promising target of protecting neurons from cerebral I/R injury.

## Introduction

Ischemic stroke (IS) is a sudden medical condition in which little or restricted blood flow to the brain results in cell death. IS has high mortality, in which almost half of the patients live less than 1 year, and high morbidity, with up to 75% of patients having physical, mental, and emotional disability (Hankey, 2017; Benjamin et al., 2018). Some mechanisms have been found to be responsible for post-IS neuronal injury, such as excitatory neurotransmitter release, calcium overload and free radical injury. However, few therapies have been discovered to treat IS efficiently (Pandya et al., 2011; Chen et al., 2012). The exact molecular pathways underlying the ischemic reperfusion (I/R) neuronal injury have not been fully illustrated.

Homer proteins are best known as scaffold proteins located at the postsynaptic density and are crucial in regulating neuronal signals (Huang et al., 2008). Homer1a, a short form of Homer protein, contains a conserved EVH1 domain but lacks the CC domain, which enables Homer1a to function as a negative regulator of long forms of Homer protein (Foa and Gasperini, 2009). Several studies have shown that Homer1a plays an important role in sleep management, anti-depression and chronic inflammatory pain (Tappe et al., 2006; Serchov et al., 2016; Diering et al., 2017). Our previous study showed that Homer1a protects neurons against N-Methyl-D-aspartate (NMDA)-induced injury (Wang et al., 2015), and we also found that Homer1a protects retinal ganglion cells against retinal I/R injury (Fei et al., 2015). However, the exact role of Homer1a in cerebral I/R injury and the associated mechanism have not been elucidated.

Mitochondria play pivotal roles in supplying cellular energy, signaling, cellular differentiation, and cell death, as well as maintaining control of the cell cycle and cell growth (Schumacker et al., 2014). As an indispensable energy reservoir, mitochondria control the lifeline of most cells. Dysfunction of mitochondria plays a key role in many nervous system diseases such as stroke and neurodegenerative diseases (Prentice et al., 2015). Studies have revealed the pivotal role of mitochondria in aggravating reperfusion injury *via* producing excessive free radicals (Sanderson et al., 2013). Many studies have focused on therapies such as antioxidants and pro-biogenesis targeting mitochondria, with substantial progress, indicating the importance of mitochondria in I/R injury (Mailloux, 2016; Bhatti et al., 2017). The endoplasmic reticulum (ER) is also an important organelle that is responsible for protein folding and calcium homeostasis. Misfolded protein overload under various stresses results in ER turbulence, which is also known as ER stress (Chen et al., 2017). Previous studies have shown that ER stress is a major mechanism in I/R injury-induced apoptosis in the liver, myocardium and brain (Wang et al., 2016; Imarisio et al., 2017; Wu et al., 2017). Our previous studies showed that Homer1a preserves mitochondrial function in PC12 cells under oxidative stress induced by hydrogen peroxide and regulates the calcium equilibrium in HT22 cells under glutamate-induced injury (Luo et al., 2012a; Rao et al., 2016). However, the underlying mechanism between Homer1a and mitochondria and whether Homer1a influences ER stress after cerebral I/R are still unclear.

In the present study, it is aimed to survey the expression dynamics of Homer1a after neuronal ischemia-reperfusion injury and to determine the effect of Homer1a against IR injury. The effect of Homer1a on mitochondria dysfunction and ERS, as well as the relationship between them are also investigated. Furthermore, drug administration is implemented to inquire the specific mechanism involving PKR-like ER kinase (PERK) and inositolrequiring protein 1 (IRE1) pathways.

## Materials and Methods

### Rat Primary Cultured Cortical Neurons

All procedures and animal manipulations were designed according to the National Institute of Health (NIH) Guide for the Care and Use of Laboratory Animals (No. 85-23) and were approved by the Ethics Committee of the Fourth Military Medical University. Primary cultured neurons were sampled from pregnant C57 mice using specified methods. Briefly, we removed embryos at 15–16 days and subsequently minced the meninges and vessels under a microscope. Then, we separated the cortex from the cerebrum and shredded it. The cortex then was digested in pancreatic enzymes for 20 min at 37°C with gentle shaking every 5 min. The digested neurons were resuspended in NM medium composed of neurobasal medium, 0.5 mM L-glutamine and 2% B27 before being plated onto the Poly-L-Lysine (PLL) pretreated dishes with a density of 1.5 × 10^6^ cells/cm^2^. The neurons were cultured in a 5% CO_2_ incubator at 37°C, and the culture medium was renewed every 3 days.

### Lentivirus Construction and Transfection

The preparation of a Homer1a Lentivirus was described previously (Luo et al., 2012a). The lentivirus overexpression open reading frame was the Pgc-fu-Homer1a cDNA-3FLAG construct (GeneChem Co., Shanghai, China). The primary cultured cortical neurons were cultured 8 days before being transfected with lentivirus for 72 h with an approximate multiplicity of infection (MOI) of 20.

### Oxygen and Glucose Deprivation (OGD)

To mimic the I/R injury, the culture medium was renewed with glucose-free Neurobasal after being washed three times with PBS, and the neurons were then cultured in a specific chamber containing 5% CO_2_ and 95% N_2_ at 37°C for 1 h to simulate ischemic injury. Neurons were then removed from the chamber and washed with PBS three times before being reinfused with NM medium and maintained in a 5% CO_2_ incubator at 37°C for a designated time to simulate the reperfusion injury.

### Cell Viability Assay

The neurons with or without various interventions were cultured with the Cell Counting Kit-8 (CCK-8; Dojindo, Japan) for 4 h at a concentration of 10 μl/5,000 neurons. The absorbance of each well was determined by a microplate reader (Bio-Rad, Hercules, CA, USA) at 450 nm.

### Lactate Dehydrogenase (LDH) Assay

A Cytotoxicity Detection Kit Plus (Roche Applied Bioscience, Indianapolis, IN, USA) was used to measure the release of lactate dehydrogenase (LDH), and all procedures were implemented following the manufacturer’s protocol. Each group had its own maximum release group, and the LDH release was calculated in each group as the experimental value/maximum release × 100%.

### Antibodies and Reagents

The primary antibodies used are listed as follows: anti-cytochrome c (1:1,000, 10993-1-AP, Proteintech, rosemont, IL, USA), anti-caspase-9 (1:1,000, #9508, Cell Signaling, Danvers, MA, USA), anti-PERK (1:1,000, #3192, Cell Signaling, Danvers, MA, USA), anti-phosphorylated-PERK (anti-p-PERK; Thr982;1:800, DF7576, Affinity Biosciences, Cincinnati, OH, USA), anti-IRE1 (1:500, ab37073, Abcam, Cambridge, MA, USA), anti-p-IRE1 (phosphor S724; 1:1,000, ab48187, Abcam, Cambridge, MA, USA), anti-activating transcription factor 6 (anti-ATF6; 1:1,000, ab203119, Abcam, Cambridge, MA, USA), anti-N’ATF6 (1:800, ab37149, Abcam, Cambridge, MA, USA) and anti-β-actin (1:3,000, GTX109639, GeneTex, Irvine, CA, USA). The ER stress activator Tunicamycin (TM) and the p-PERK inhibitor GSK2656157 were obtained from MedChem Express (Monmouth Junction, NJ, USA). The p-IRE1 inhibitor 3,5-dibromosalicylaldehyde (DBSA) was obtained from Tokyo Chemical Industry (TCI, Tokyo, Japan).

### Western Blot Analysis

Neurons were lysed by RIPA with a protease inhibitor cocktail (Roche Applied Bioscience, Indianapolis, IN, USA), and the protein concentration of each sample was measured using a BCA quantification assay (Biosynthesis biotechnology, Beijing, China). Equivalent amounts of protein (30 μg) were separated by 6%–12% SDS-PAGE gels before being transferred onto the methanol pretreated polyvinylidene difluoride (PVDF) membranes. After being soaked in 5% defatted milk dissolved in Tris-phosphate buffer with 0.05% Tween 20 (TBST) for 1 h, the membranes were incubated overnight at 4°C. The membrane was incubated in secondary antibody diluent for 1 h at room temperature before its immunoreactivity was detected using Super Signal West Pico Chemiluminescent Substrate (Thermo Scientific, Rockford, IL, USA). The intensity of the protein bands was calculated *via* ImageJ with normalization to that of β-actin.

### Cytoplasmic/Mitochondrial Protein Extraction

This procedure was implemented by using ProteoExtract Cytosol/Mitochondria Fractionation Kit (Calbiochem, Germany) and subjected to the described protocols. Briefly, the neurons were collected with Accutase (Invitrogen, Carlsbad, CA, USA) and centrifuged twice with PBS at 600 *g* for 5 min. Then, the 1× cytoplasmic extraction buffer was used to resuspend the neurons on ice for 10 min before homogenizing the neuron suspension, which was then centrifuged at 700 *g* for 10 min at 4°C. The cytoplasmic protein was obtained by collecting the supernatant after centrifuging the supernatant collected in the last step at 10,000 *g* for 30 min at 4°C. The mitochondrial protein was obtained by resuspending the sediment with mitochondrial extraction buffer and vortexing for 10 s.

### Measurement of Reactive Oxygen Species (ROS) Production

The intracellular reactive oxygen species (ROS) was determined using 2,7-dichlorodihydrofluorescein diacetate (H2-DCFDA), an intracellular ROS indicator, as previously reported. Cultured cortical neurons were incubated with 10 nM H2-DCFDA for 1 h at 37°C in a dark place. Then, the neurons were resuspended with PBS before being read by a fluorescence plate reader (excitation wavelength of 480 nm, emission wavelength of 530 nm).

### Measurement of Mitochondrial Membrane Potential (MMP)

To monitor the mitochondrial membrane potential (MMP) of each group, the neurons were incubated in 10 mM fluorescent dye rhodamine 123 (RH 123) for 30 min at 37°C. Then, the neurons were washed with PBS three times before reading the fluorescence with a fluorescence plate reader (excitation wavelength of 480 nm, emission wavelength of 530 nm).

### Measurement of Intracellular ATP

The ATP level was intraneuronally evaluated using an ATP assay kit (Beyotime, Shanghai, China), and the procedures were performed following the manufacturer’s protocol. Briefly, the culture medium in the 96-well plate was removed, and the neurons were resuspended with 100 μl of working solution (ATP detection reagent: diluent = 1:5) for 5 min at room temperature. The ATP indication of each group was obtained *via* a fluorospectrophotometer and modified as a percentage of the control.

### Immunofluorescence Staining

Cultured neurons were fixed in 4% paraformaldehyde for 20 min at room temperature in a dark place. Then, the neurons were treated with 0.3% Triton-X-100 for 20 min and blocked by 10% donkey serum for 3 h. After being washed with 1× PBS three times, the neurons were then incubated with rabbit anti-p-PERK (1:200) or rabbit anti-p-IRE1 (1:200) at 4°C overnight. The DAPI nuclei-staining was implemented for 10 min after the incubation of Alexa 594 donkey-anti-rabbit IgG (1:400) for 2 h at room temperature. The neurons were then visualized with a fluorescence microscope. To compare the immunoreactivity between different groups, all images were collected using the identical laser power, exposure time and detector sensitivity. At least five images were acquired by an researcher who was blinded to the experimental details.

### Statistical Analysis

All the experimental procedures were performed at least three times. The statistical analysis was conducted using Bonferroni’s multiple comparisons or an unpaired *t*-test (two groups) and univariate analyses of variance (ANOVA; more than two groups); the results were plotted with GraphPad Prism Software 6.0.

## Results

### Homer1a Protects Neurons Against I/R Injury

**Figure 1:**
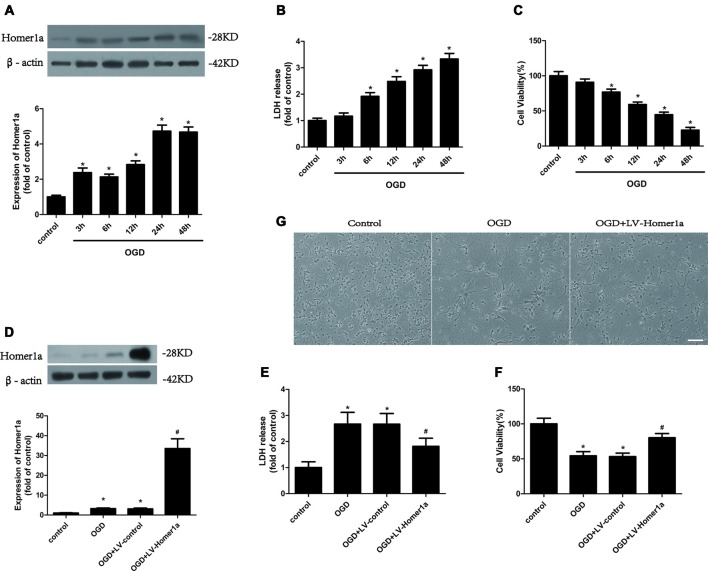
Homer1a protects neurons against ischemia/reperfusion (I/R) injury. Cultured cortical neurons were treated with oxygen and glucose deprivation (OGD) for 1h, and the expression level of Homer1a protein at different reperfusion time points were investigated by Western blot **(A)**. Then the cytotoxicity of neurons was detected by lactate dehydrogenase (LDH) release assay **(B)** and the cell viability was evaluated by CCK-8 test **(C)**. The cortical neurons were then transfected with LV-Homer1a or LV-control for 72 h and exposed to OGD. The Homer1a protein expression was detect by Western blot **(D)**, and cytotoxicity and cell viability were tested in each group **(E,F)**. The cortical neurons were also observed under representative phase photomicrographs **(G)**. The data are represented as means ± SEM. *p < 0.05 vs. Control, ^#^*p* < 0.05 vs. OGD.

To determine the effect of I/R injury on Homer1a, the cultured cortical neurons were treated with oxygen and glucose deprivation (OGD) for 1 h, and the expression level of Homer1a protein at different reoxygenation time points was investigated using Western blot. The results showed that the Homer1a protein levels increased significantly at 6, 12, 24 and 48 h with reperfusion (Figure 1A). The I/R injury-induced LDH release (Figure 1B) and reduced cell viability of neurons (Figure 1C) were elevated in a time-dependent manner. Given that the neuron survival was almost 50%, a 24 h reperfusion after OGD was used in the following experiments. To evaluate the effects of Homer1a, the Homer1a-targeted lentivirus (LV-Homer1a) or the negative control lentivirus (LV-control) was transfected into the neurons for 72 h, and the Western blot results showed that the LV-Homer1a protein expression was significantly increased (Figure 1D). The overexpression of Homer1a clearly decreased LDH release (Figure 1E) and promoted cell viability (Figure 1F) after I/R injury. Furthermore, representative phase photomicrographs exhibited that the LV-Homer1a transfected neurons showed less cell death relative to neurons without LV-Homer1a transfection (Figure 1G).

### Homer1a Conserves Mitochondrial Function in Neurons after I/R Injury

**Figure 2:**
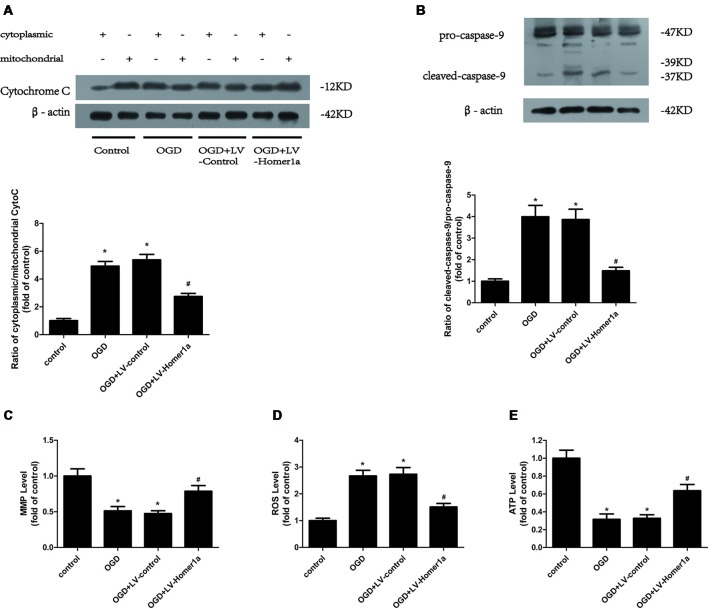
Homer1a conserves mitochondrial function in neurons after I/R injury. Cultured cortical neurons were transfected with LV-Homer1a or LV-control for 72 h and exposed to OGD. The cytochrome c release to cytoplasm and the activation of caspase-9 were detected by Western blot **(A,B)**. The mitochondrial membrane potentials (MMPs) were detected by Rh123 test **(C)**, the intracellular reactive oxygen species (ROS) level was investigated through H_2_-DCFDA **(D)** and the intracellular ATP level was measured by ATP assay **(E)**. The data are represented as means ± SEM. *p < 0.05 vs. Control, ^#^*p* < 0.05 vs. OGD.

We also evaluated whether Homer1a conserves the mitochondrial function following I/R injury. We transfected LV-Homer1a 72 h before exposing cultured cortical neurons to the OGD and reperfusion. The results revealed that the overexpression of Homer1a clearly decreased the cytochrome c release from the mitochondria to the cytoplasm (Figure 2A) and caspase-9 activation (Figure 2B). Additionally, Homer1a expression alleviated MMP loss (Figure 2C), reduced the intracellular ROS level (Figure 2D) and improved ATP loss (Figure 2E) in neurons that underwent I/R injury compared with that in neurons without LV-Homer1a transfection.

### Homer1a Reduces ER Stress in Neurons That Underwent I/R Injury

**Figure 3:**
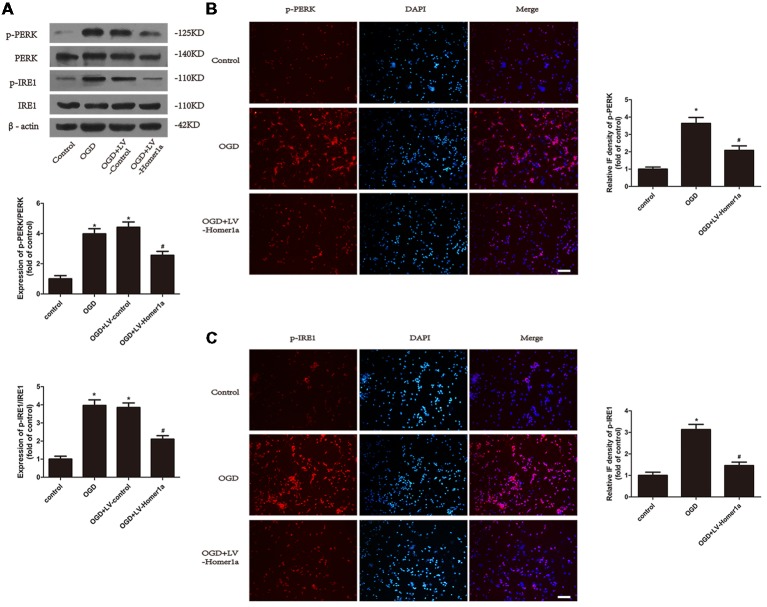
Homer1a reduces endoplasmic reticulum (ER) stress in neurons that underwent I/R injury. Cultured cortical neurons were transfected with LV-Homer1a or LV-control for 72 h and exposed to OGD. Levels of phosphorylated-PKR-like ER kinase (p-PERK), PERK, phosphorylated-IRE1 (p-IRE1) and IRE1 were measured with Western blot **(A)**. The level of p-PERK and p-IRE1 were also determined with immunofluorescence (IF) staining. Images were captured with fluorescence microscope. Red: p-PERK or p-IRE1, blue: nuclei-staining by DAPI. Scale bar = 50 μm. The IF intensity of each group was calculated with ImageJ **(B,C)**. The data are represented as means ± SEM. *p < 0.05 vs. Control, ^#^*p* < 0.05 vs. OGD.

We next examined the effect of Homer1a on ER stress after I/R exposure. The Western blot results showed that under the I/R injury, the ratios of p-PERK/PERK and p-IRE1/IRE1 were both reduced in neurons with Homer1a overexpression compared with neurons without LV-Homer1a lentivirus transfection (Figure 3A), whereas the Homer1a overexpression showed little effect on expression of ATF6 and its active form N’ATF6 (Supplementary Figure S1). The immunofluorescent results indicated that the effect of I/R injury on neurons significantly increased the fluorescence intensity of p-PERK and p-IRE1. However, neurons with Homer1a overexpression exhibited a significantly decreased fluorescence intensity of p-PERK and p-IRE1 (Figures 3B,C). Together, the above data showed that Homer1a overexpression reduced IR-induced ER stress.

### Homer1a Alleviates Mitochondria Dysfunction by Reducing ER Stress After I/R Injury

**Figure 4:**
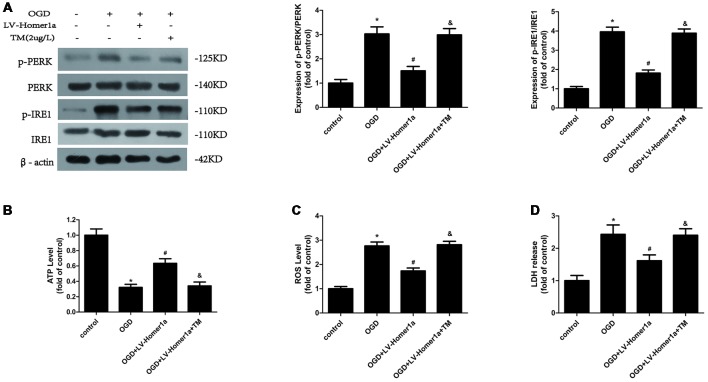
Homer1a alleviates mitochondria dysfunction by reducing ER stress after I/R injury. Cultured cortical neurons were pre-treated with 2 μg/L Tunicamycin (TM) 24 h to activate the ER stress and exposed to I/R injury. The effects of TM were tested by Western blot **(A)**. The intracellular ATP level was measured by ATP assay **(B)** and the intracellular ROS level was investigated through H_2_-DCFDA **(C)**. We also investigated the neurons cytotoxicity of each group with LDH release assay **(D)**. The data are represented as means ± SEM. *p < 0.05 vs. Control, ^#^*p* < 0.05 vs. OGD, ^&^*p* < 0.05 vs. OGD+LV-Homer1a.

To test whether the Homer1a effect of ER stress reduction is responsible for the conservation of mitochondrial function, we pretreated cortical neurons with 2 μg/L TM, an ER stress activator, 24 h before exposure to the I/R injury to counteract the ER stress reduction of Homer1a overexpression. The Western blot results showed that the TM abolished the reduced p-PERK/PERK and p-IRE1/IRE1 due to Homer1a overexpression (Figure 4A). Furthermore, the TM pretreatment of cortical neurons with LV-Homer1a-transfection under I/R injury reversed the ATP loss reduction (Figure 4B) and intracellular ROS reduction (Figure 4C) achieved by Homer1a overexpression. Additionally, under I/R injury, LDH release was greater in neurons with LV-Homer1a and TM than in neurons with LV-Homer1a alone (Figure 4D).

### Homer1a Preserves Mitochondrial Function by Reducing ER Stress Mainly *via* the PERK Pathway After I/R Injury

**Figure 5:**
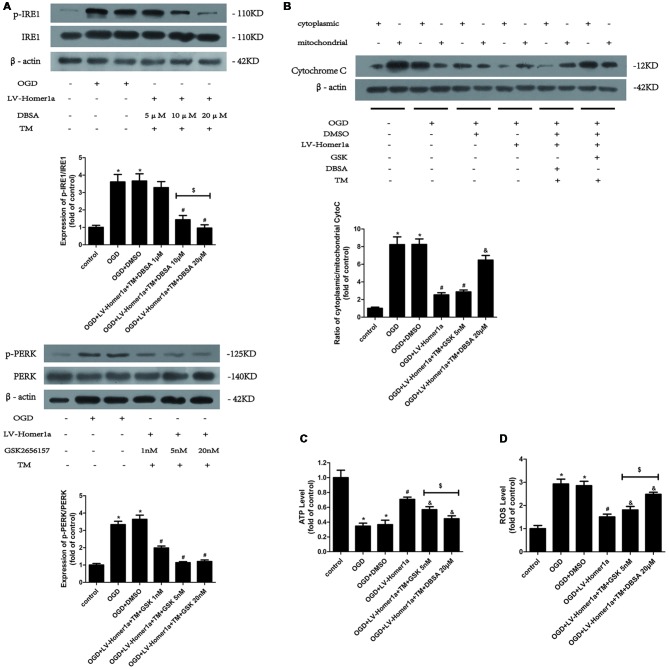
Homer1a preserves mitochondrial function by reducing ER stress mainly *via* the PERK pathway after I/R injury. TM was used to activate the ER stress whereas the GSK2656157 and 3,5-dibromosalicylaldehyde (DBSA) were used to inhibit the PERK and IRE1 respectively. Different DBSA concentrations were tested in cortical neurons and detected by Western blot **(A)** as well as GSK2656157 **(B)**. Then 20 μM DBSA and 5 nM GSK2656157 were chosen to apply to follow experiments. The cytochrome c release to cytoplasm in each group was detected through calculating the ratio of cytoplasmic/mitochondrial cytochrome c by Western blot **(B)**. The intracellular ATP level was measured by ATP assay **(C)** and the intracellular ROS level was investigated through H_2_-DCFDA **(D)**. The data are represented as means ± SEM. ^$^*p* < 0.05, *p < 0.05 vs. Control, ^#^*p* < 0.05 vs. OGD, ^&^*p* < 0.05 vs. OGD+LV-Homer1a.

To further elucidate the mechanisms by which Homer1a reduces the ER stress to preserve mitochondrial function, we pretreated cortical neurons 24 h before any intervention with specific inhibitors to separately test each pathway of ER stress. As the Western blot results showed, 20 μM p-IRE1 specific inhibitor DBSA effectively blocked the phosphorylation of IRE1 (Figure 5A), and 5 nM GSK2656157, a specific p-PERK inhibitor, was efficient in reducing the phosphorylation of PERK (Figure 5B). As the appropriate dosage of each inhibitor was employed, the Western blot results revealed that neurons pretreated with TM and GSK2656157 showed significant reductions in cytochrome c release from mitochondria to the cytoplasm, whereas neurons pretreated with TM and DBSA showed little change under I/R injury (Figure 5C). Furthermore, compared with neurons pretreated with TM and DBSA, neurons pretreated with TM and GSK2656157 exhibited less ATP loss and a lower intracellular ROS level (Figure 5D).

## Discussion

The involvement of Homer1a in some central neuronal stress conditions has been found in our and previous studies (Luo et al., 2014; Schumacker et al., 2014; Wu et al., 2018). In this study, we revealed that Homer1a protects against neuronal I/R injury by preserving mitochondrial function and regulating ERS in primary cortical neurons. First, upregulated expression of Homer1a alleviated the I/R injury with less neuronal death. Second, upregulation of Homer1a helps restore mitochondrial function and mitigates ER stress activation. Third, the anti-ER stress effects of Homer1a were partially responsible for its pro-mitochondrial roles, which was attained mainly through the PERK pathway.

As an immediate early gene (IEG) that regulates the longer form of the homer gene, Homer1a was minimally expressed in the brain and other areas of the nervous system until the physiological conditions were disturbed. Studies have shown that upregulated Homer1a expression evoked by various forms of stress could help fight against various pathological processes (Tappe et al., 2006; Diering et al., 2017; Leber et al., 2017). Our previous study has shown that Homer1a protects neurons against TNF-a induced injury by interacting with the MAPK pathways (Luo et al., 2012b). Additionally, Wang et al. (2015). found the protective effect of Homer1a against NMDA-induced injury (Rao et al., 2016). Recently, Wu et al. (2018) found that Homer1a contributed a protective effect in HT22 cells under oxidative stress by inducing autophagy. Similarly, our study demonstrated that Homer1a was minimally expressed in rat cortical neurons but rose quickly after the onset of the I/R injury in a time-dependent manner that tended to dynamically stabilize after 24 h of reperfusion. Overexpression of Homer1a with a specific lentivirus increased neuronal survival and decreased LDH release after OGD, which indicated that Homer1a plays a protective role in the neuronal system in I/R conditions.

Because of the high demand for energy, the nervous system is more susceptible to mitochondrial dysfunction that impaired mitochondria is incapable of producing enough ATP to fill neuronal needs (Shulyakova et al., 2017). ROS production by mitochondria occurs in both physiological and pathological processes, and mitochondria dysfunction leads to the burst of ROS production and hinders oxidative phosphorylation as the mitochondrial membrane breaks down and finally leads to apoptotic cell death (Schieber and Chandel, 2014; Martin et al., 2018). Furthermore, the weakened membrane with higher permeability could lead to apoptotic factor release, such as the release of apoptosis-inducing factor and cytochrome c (Niizuma et al., 2010; Leaw et al., 2017). Cytochrome c includes procaspase-9, which is cleaved into caspase-9 and further triggers neuron apoptosis (Bennetts and Pierce, 2010). Our previous studies showed that Homer1a overexpression helps reduce oxidative stress and restore mitochondrial function in PC12 cells and HT-22 cells under oxidative stress (Luo et al., 2012a; Rao et al., 2016). Consistent with these studies, our results showed that Homer1a overexpression reduces ROS production and attenuates ATP and MMP loss in cortical neurons. Additionally, the overexpression of Homer1a reduces cytochrome c release from mitochondria to the cytoplasm as the ratio of cytoplasmic/mitochondrial cytochrome c decreases, which indicates that the Homer1a overexpression prevented the mitochondria from permeabilizing. The apoptic cell death was alleviated as the caspase-9 activation was reduced after Homer1a expression. All these results provide evidence that Homer1a protects mitochondrial function under I/R injury and thus stops neuronal apoptosis.

Three major transmembrane proteins are involved in the ER stress-activated unfolded protein response (UPR): IRE1, PERK and ATF6. All three proteins are coupled with the chaperone protein Bip and stay inactive under physiological conditions. When the UPR is activated, after the Bip dissociation, the IRE1 and PERK oligomerize and phosphorylate to activate their downstream signals, and ATF6 is cleaved by the Golgi and moves into the nucleus to act as a transcription promoter (Darling and Cook, 2014). ER stress is activated after IS and leads to cell damage (Yu et al., 2017). In our study, the results showed that IR significantly activated ER stress as evidenced by the hyperphosphorylation of PERK and IRE1 and enhanced fluorescence intensity of p-PERK and p-IRE1. Our previous study showed that downregulation of Homer1b/c, the long form in the homer family that plays an opposite role as Homer1a, inhibited the expression of CHOP, a downstream gene of all three pathways of ER stress, is considered to be a pro-apoptotic protein that mediates ER stress-induced apoptosis (Chen et al., 2012). Similarly, in the present study, our Western blot results showed less phosphorylation of the IRE1 and PERK proteins and reduced fluorescence intensity of phosphorylated IRE1 and PERK. These results demonstrate the anti-ER stress roles of Homer1a. However, our results showed no significant relationship between ATF6 and Homer1a overexpression which may due to the Homer1a itself, our stroke model, lab environment or other specific details. ATF6 pathway is one of the main branches of ER stress and its activation could upregulates activating transcription factor 4 (ATF4), a cAMP-response element binding protein that could cause retinal and neuronal degeneration (Gully et al., 2016), and CHOP expression (Darling and Cook, 2014). Besides, ATF6 has two isoforms that may be crucial in many scenarios such as embryogenesis (Thuerauf et al., 2007). It is necessary to carry out further experiments to elucidate ATF6’s specific roles in brain I/R injury.

ER stress plays different roles in different situations. Under acute or chronic pathologies or stress, ERS elicits UPR, which facilitates the misfolding protein degradation that helps alleviate protein overload that, in turn, helps to avoid cell apoptosis. However, when the cell proteostasis is still not restored, ERS can trigger apoptosis *via* several pathways (Tabas and Ron, 2011). This bifunctional process is essential for maintaining cell homeostasis. Many studies have shown that ERS activation could further aggravate the original pathology, such as pancreatic disease, intestinal inflammation and liver injury (Cunha et al., 2009; Han et al., 2016; Hosomi et al., 2017). In IS, the inhibition of ERS alleviates neuronal injury (Feng et al., 2017). Only a few studies have shown that the activation of ERS protects cells from apoptosis (Tan et al., 2017). In our *in vitro* model, aggravated injury, as demonstrated by increased LDH release, was found in TM-treated neurons. The results indicated that the ERS plays pro-apoptotic roles in cortical neurons under I/R injury.

ER and mitochondria both have dynamic plasticity of their structure in response to cellular requirements, which is the prerequisite for ER-mitochondrial communication functionally and structurally (Bravo-Sagua et al., 2013). The activation of ER stress induced the mitochondrial dysfunction in HT22 cells (Xu et al., 2018), and ER stress sensitized the mitochondria by opening its permeability transition pore in cardiac injury (Chen et al., 2017). Consistent with these studies, our results demonstrate that in cortical neurons under I/R injury, the reactivation of Homer1a overexpression suppressed ER stress *via* TM, which led to increased intracellular calcium load, elevated ROS production and ATP loss. This finding indicated that Homer1a conserves mitochondrial function by inhibiting ER stress under I/R injury in cortical neurons. On the other hand, mitochondria may also have effects on ER stress. Studies showed that the mitochondria and ER stress has crosstalk in many models (Malhotra and Kaufman, 2011; Senft and Ronai, 2015), and the block of respiratory chain complex with rotenone or antimycin A that leads to mitochondrial dysfunction exerts great effects on ER stress (Lee et al., 2010; Jeon et al., 2017; Heo et al., 2019). Further studies are needed to investigate the effects of mitochondria to ER stress regarding Homer1 protein regulation.

**Figure 6:**
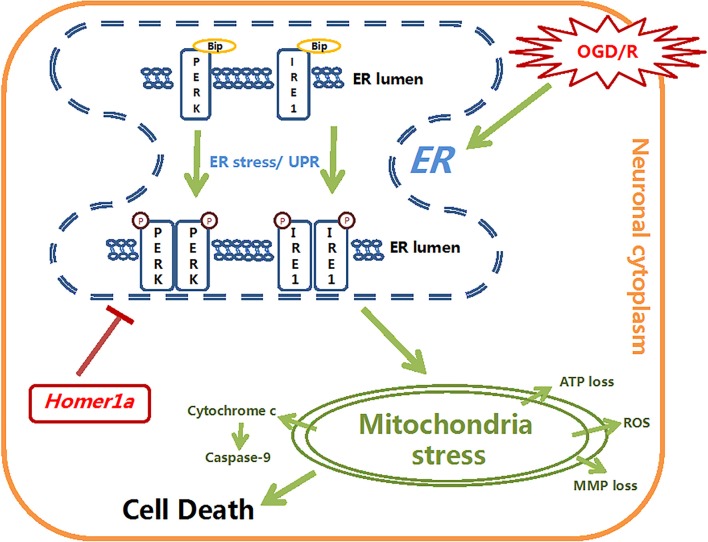
The schematic diagram depicts the molecular mechanisms of homer1a-induced neuron-protection against OGD injury. Homer1a overexpression inhibited the OGD-induced activation of ER stress, which alleviated the secondary mitochondria dysfunction by regulating ATP, MMP, ROS, cytochrome c and caspase-9. In addition, Homer1a-induced preservation of mitochondrial function was achieved mainly *via* inhibiting the PERK pathway.

Additionally, we further investigated the mechanism by which Homer1a exerts mitochondrial protection *via* ER stress reduction. According to previous studies, the phosphorylated PERK can activate ATF4 transcription, which in turn promotes the expression of bZIP transcription factor C/EBP homologous protein (CHOP) and then influences mitochondrial function by hyperoxidizing the ER lumen structure by activating ER oxidase 1α (ERO1α; Harding et al., 2000; Song et al., 2008). The oxidized ER activates IP3R-induced calcium release and then activates Ca/calmodulin-dependent protein kinase II (CaMKII), which facilitates calcium transfer into the mitochondria. The increasing mitochondrial calcium uptake leads to MMP loss, cytochrome c release and apoptosis (Kashiwase et al., 2005; Li et al., 2009; Timmins et al., 2009). In this study, compared with neurons with Homer1a overexpression, neurons in which the PERK pathway is activated *via* TM and p-IRE1 inhibitor DBSA under I/R injury showed more cytochrome c release, elevated ATP loss and an increased ROS level, which indicated that the PERK pathway is involved in mitochondrial dysfunction. Previous studies also found that in gastric cancer cells, the phosphorylated IRE1 activated Jun N-terminal protein kinase-1 (JNK-1), which in turn activated the transcription of CHOP and influenced mitochondrial related functions (Kim et al., 2018). However, in our study, activating the IRE1 pathway in neurons under I/R injury with TM and the PERK inhibitor GSK2656157 showed less abortion of Homer1a protection with less ATP loss and ROS production than PERK pathway activation. There was no significant difference in cytochrome c release between neurons in which ER stress was inhibited by Homer1a lentivirus transfection and neurons in which IRE1 was activated by TM and GSK2656157. These discrepancies may be attributed to different models, unknown biological traits of the inhibitors and a potential interaction between Homer1a and IRE1-JNK related pathways. All these results indicate that Homer1a preserves mitochondrial function mainly through the PERK pathway and partially through the IRE1 pathway (Figure 6).

A limitation of this study was that Homer1a’s effect on mitochondria dysfunction and ER stress under I/R injury was only explored in cultured cortical neurons, lacking the interaction with glial cell signaling. Our lab has already built a Homer1a knockout mouse model in which more experiments will be implemented to further evaluate these observations. Additionally, in this study, we investigated molecular mechanisms in pharmacological ways, and future studies with specific gene modulation should elucidate the exact mechanism of Homer1a in mitochondria protection.

In this study, the primary cultured neurons may contain a small portion of astrocytes and may insert mutual or extra efforts on neuron outcome with OGD/R model. Researches revealed that the astrocyte could protect neurons from I/R injury (Liu et al., 2018), while some suggested that the astrocyte plays harmful roles in IS (Yang et al., 2017). Since the ER stress inhibition *via* Homer1a overexpression could inhibit astrocyte activation (Huang et al., 2017), the roles of astrocyte in our present study are still unclear. Further experiments are needed to find the exact roles of astrocyte in IS.

In conclusion, we suggested that Homer1a may protect against I/R neuronal injury by preserving mitochondrial function though suppressing ER stress. Interestingly, Homer1a’s protection of mitochondrial function is mainly by inhibiting the PERK pathway. Taken together, our findings provide novel perceptions about the relationship between Homer1a, ER stress and mitochondrial dysfunction in neurons under I/R conditions.

## Author Contributions

ZF conceived the idea and designed the experiments. JW and XW carried out most of the experiments and composed the manuscript. PL and KY carried out partial experiments. YY and JP helped in analyzing experimental results. LZ and SD assisted with verifying the results. DH helped revising the manuscript.

## Conflict of Interest Statement

The authors declare that the research was conducted in the absence of any commercial or financial relationships that could be construed as a potential conflict of interest.

## Supplementary Material

FIGURE S1 Relationship between Homer1a and ATF6 pathway after I/R injury of neurons. Cultured cortical neurons were transfected with LV-Homer1a or LV-control for 72 h and exposed to OGD. Levels of ATF6 and N’ATF6 were measured with Western blot. The data are represented as means ± SEM. **p* < 0.05 vs. Control.
