# Supplementary figures and images for: Homer1a Attenuates Endoplasmic Reticulum Stress-Induced Mitochondrial Stress After Ischemic Reperfusion Injury by Inhibiting the PERK Pathway
Source: Front Cell Neurosci. 2019 Mar 15;13:101. doi: 10.3389/fncel.2019.00101 (PMC6428733; doi:10.3389/fncel.2019.00101)

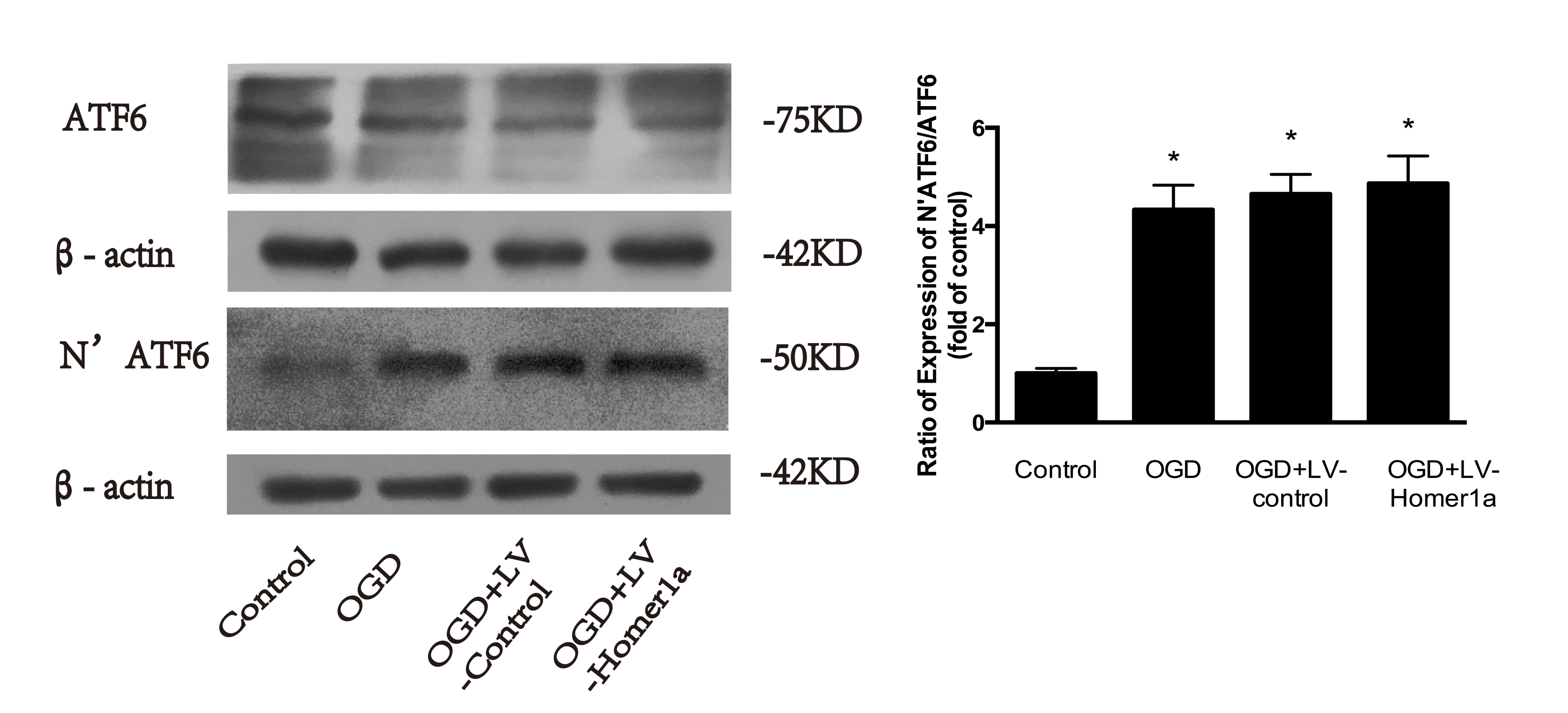

Supplement: FIGURE S1 — Relationship between Homer1a and ATF6 pathway after I/R injury of neurons. Cultured cortical neurons were transfected with LV-Homer1a or LV-control for 72 h and exposed to OGD. Levels of ATF6 and N’ATF6 were measured with Western blot. The data are represented as means ± SEM. *p < 0.05 vs. Control. [file Image_1.TIF]
